# Current News

**Published:** 1903-09

**Authors:** 


### Current News.

### NEW JERSEY STATE BOARD OF REGISTRATION AND EXAMINATION IN DENTISTRY.

The State Board of Registration and Examination in Dentistry of New Jersey will hold its semiannual examination in the Assembly-room of the State-House at Trenton, N. J., on the 20th, 21st, and 22d of October, 1903.

All applicants are required to file their applications with the secretary ten days prior to the examination.

Sessions begin promptly at 9 A.M. each day.

For information in regard to examinations communicate with the secretary, Charles A. Meeker, D.D.S., 29 Fulton Street, Newark, N. J.

Fee for examination $25.00.

Chas. A. Meeker,
*Secretary Dental Commission.*

---

### AMERICAN SOCIETY OF ORTHODONTISTS.

The third annual meeting of the American Society of Orthodontists will be held on December 30 and 31, 1903, and January 1, 1904, in Buffalo, N. Y.

Anna Hopkins,
*Secretary.*

St. Louis, Mo.

